# Revisiting *Vitis vinifera* Subtilase Gene Family: A Possible Role in Grapevine Resistance against *Plasmopara viticola*

**DOI:** 10.3389/fpls.2016.01783

**Published:** 2016-11-25

**Authors:** Joana Figueiredo, Gonçalo J. Costa, Marisa Maia, Octávio S. Paulo, Rui Malhó, Marta Sousa Silva, Andreia Figueiredo

**Affiliations:** ^1^Biosystems & Integrative Sciences Institute, Faculdade de Ciências, Universidade de LisboaLisboa, Portugal; ^2^Laboratório de FTICR e Espectrometria de Massa Estrutural, Faculdade de Ciências, Universidade de LisboaLisboa, Portugal; ^3^Centro de Química e Bioquímica, Faculdade de Ciências, Universidade de LisboaLisboa, Portugal; ^4^Computational Biology and Population Genomics Group, Centre for Ecology, Evolution and Environmental Changes, Faculdade de Ciências, Universidade de LisboaLisboa, Portugal

**Keywords:** subtilases, *Vitis vinifera*, *Plasmopara viticola*, immunity, gene expression

## Abstract

Subtilisin-like proteases, also known as subtilases, are a very diverse family of serine peptidases present in many organisms. In grapevine, there are hints of the involvement of subtilases in defense mechanisms, but their role is not yet understood. The first characterization of the subtilase gene family was performed in 2014. However, simultaneously, the grapevine genome was re-annotated and several sequences were re-annotated or retrieved. We have performed a re-characterization of this family in grapevine and identified 82 genes coding for 97 putative proteins, as result of alternative splicing. All the subtilases identified present the characteristic S8 peptidase domain and the majority of them also have a pro-domain I9 inhibitor, a protease-associated (PA) domain, and a signal peptide for targeting to the secretory pathway. Phylogenetic studies revealed six subtilase groups denominated *VvSBT*1 to *VvSBT*6. As several evidences have highlighted the participation of plant subtilases in response to biotic stimulus, we have investigated subtilase participation in grapevine resistance to *Plasmopara viticola*, the causative agent of downy mildew. Fourteen grapevine subtilases presenting either high homology to P69C from tomato, SBT3.3 from *Arabidopsis thaliana* or located near the Resistance to *P. viticola* (RPV) locus were selected. Expression studies were conducted in the grapevine-*P. viticola* pathosystem with resistant and susceptible cultivars. Our results may indicate that some of grapevine subtilisins are potentially participating in the defense response against this biotrophic oomycete.

## Introduction

Subtilisin-like proteases (SBTs) are the second largest family of serine peptidases present in archaea, bacteria, eukarya, fungi, and yeast (Siezen et al., 1991). They belong to the S8 family within the SB clan of serine proteases, according to the classification of the peptidase database MEROPS (Rawlings et al., 2014; http://merops.sanger.ac.uk). The majority of plant subtilases are synthesized as an inactive pre-proprotein precursor. Their structure usually presents a signal peptide, a pro-domain (also known as I9 inhibitor domain), a subtilase domain (also known as S8 peptidase domain), and a protease-associated domain (PA), although some of them may have only one or even additional domains (Siezen and Leunissen, 1997; Dodson and Wlodawer, 1998; Antão and Malcata, 2005; Siezen et al., 2007; Vartapetian et al., 2011). The presence of a highly conserved catalytic triad within the S8 peptidase domain, composed by aspartate (Asp), histidine (His), and serine (Ser) amino acid residues is characteristic of the subtilase family (Dodson and Wlodawer, 1998). Additionally, certain subtilases may also have a conserved catalytic asparagine (Asn) residue in the same S8 peptidase domain (Siezen and Leunissen, 1997; Dodson and Wlodawer, 1998; Jordá et al., 1999). Furthermore, several plant subtilases also contain a fibronectin (Fn) III-like domain, required for their activation (Rawlings and Salvesen, 2013).

In opposition to mammals on which only nine subtilases have been identified, subtilases from plants are especially abundant, with 63 known genes in *Oryza sativa* (Tripathi and Sowdhamini, 2006), 56 genes in *Arabidopsis thaliana* (Rautengarten et al., 2005), 15 genes in *Lycopersicon esculentum* (Meichtry et al., 1999), 23 genes in the moss *Physcomitrella patens*, 90 genes in *Populus trichocarpa* (Schaller et al., 2012), and 82 genes in *Solanum tuberosum* (Norero et al., 2016). The expansion of the SBT family in plants was accompanied by functional diversification, and novel, plant-specific physiological roles were acquired in the course of evolution. In addition to their contribution to general protein turnover, plant SBTs are involved in the development of seeds and fruits, cell wall modification, processing of peptide growth factors, epidermal development, and pattern formation and in biotic and abiotic stress responses (reviewed in Schaller et al., 2012). In plant-pathogen interactions, the first evidences of subtilase participation were reported by Granell et al. (1987). This subtilase, named pathogenesis-related protein 69 (P69), was later associated to the response of tomato leaves to *Phytophothora infestans* (Christ and Mösinger, 1989; Fischer et al., 1989) and characterized as an alkaline proteinase located in the vacuole and intercellular spaces of leaf parenchyma cells (Vera and Conejero, 1988; Vera et al., 1989; Tornero et al., 1997; Jordá et al., 1999, 2000; Meichtry et al., 1999). P69 was also the first plant subtilase for which two protein substrates were identified, systemin (Schaller and Ryan, 1994) and the leucine-rich repeat protein (LRP; Tornero et al., 1996), although the consequences of these substrates processing events for plant pathogen interaction still remain unknown. More recently, Ramírez and co-workers have identified a SBT3.3 gene from *A. thaliana* as encoding a serine protease homolog to the P69C subtilase from tomato and associated its function in immune priming responses (Ramírez et al., 2013). Also, in *S. tuberosum*, expression profile analysis of detached potato leaves after *P. infestans* infection or after BABA or BTH treatment highlighted an expression increase of several subtilases genes (Norero et al., 2016). Moreover, the subtilase St SBTc-3 has been found as a major protein in apoplast of detached potato leaves after *P. infestans* infection (Fernández et al., 2012) and it was shown that this subtilase evidences DEVDasa activity and is related to programmed cell death functions (Fernández et al., 2015).

In grapevine, the first clues highlighting subtilase participation defense mechanisms were reported by Figueiredo et al. (2008), when comparing resistant and susceptible genotypes prior and post-inoculation with *Plasmopara viticola*. A subtilisin-like protease (XM_010660203.1), identified as a cucumisin was constitutively expressed in resistant genotype and increased its expression after *P. viticola* inoculation (Figueiredo et al., 2008, 2012; Monteiro et al., 2013). Also in this pathosystem, it was shown that after treatment with serine protease inhibitors, plants became more sensitive to *P. viticola* (Gindro et al., 2012). It was hypothesized that some components of *P. viticola* secretome could inhibit the endogenous subtilases of susceptible varieties, thereby inhibiting the plant's normal defense reaction, while resistant or immune varieties may possess endogenous subtilases that are not recognized due to slight structural modifications of the protein patterns of these cultivars. In this case, plant defense mechanisms would continue to operate, with fatal consequences for the pathogen and restricting its development (Gindro et al., 2012).

In 2014, a first attempt to characterize the grapevine subtilase family was made by Cao and co-workers where the subtilase sequences were identified based on the presence of the PA domain (Cao et al., 2014). In parallel, a re-annotation of the grapevine genome was conducted (Vitulo et al., 2014) and several subtilase sequences were either re-annotated or retrieved. The aim of this work was to identify subtilisin-like proteases in the grapevine genome and characterize them based on phylogenetic analyses, gene and protein primary structure. Additionally, expression analysis of selected subtilase genes was conducted to identify subtilases potentially involved in downy mildew resistance.

## Materials and methods

### Grapevine subtilase sequence retrieval and identification

In order to identify members of the grapevine subtilase gene family, the amino acid sequence of the conserved domains PA (PF02225), S8 Peptidase (PF00082), and I9 Inhibitor (PF05922) were used as query for blast searches at NCBI (http://www.ncbi.nlm.nih.gov/) and MEROPS (Rawlings et al., 2014; https://merops.sanger.ac.uk/) databases (March, 2016).

### Domain structure analysis, sequence properties, and subcellular location prediction

SBT proteins domains and active sites were analyzed in Pfam (Finn et al., 2016; http://pfam.xfam.org/) and InterProScan (Jones et al., 2014; https://www.ebi.ac.uk/interpro/search/sequence-search) databases (March, 2016); signal peptide was detected using the SignalP v4.1 (Petersen et al., 2011; http://www.cbs.dtu.dk/services/SignalP/). Molecular weight (Mw) and theoretical isoelectric point (pI) were predicted using the Protparam tool from ExPASy (Gasteiger et al., 2005; http://web.expasy.org/protparam/). Subcellular location of the subtilase proteins was predicted using TargetP v1.1 (Emanuelsson et al., 2000; http://www.cbs.dtu.dk/services/TargetP/) and PredoTar v1.3 (Small et al., 2004; https://urgi.versailles.inra.fr/predotar/predotar.html).

### Chromosomal location and gene structure

The subtilase genes were mapped in *V. vinifera* chromosomes with the Map Viewer tool from NCBI (http://www.ncbi.nlm.nih.gov/mapview/) and the blast tool at Grape Genome Browser (http://www.genoscope.cns.fr/externe/GenomeBrowser/Vitis/).

Gene exon/intron structure information was collected from grapevine genome annotation at NCBI.

The physical map constructed with grapevine subtilases gene location was also compared to a genetic linkage map representing *P. viticola* resistance (RPV) QTLs in grapevine (Merdinoglu et al., 2003; Fischer et al., 2004; Wiedemann-Merdinoglu et al., 2006; Welter et al., 2007; Bellin et al., 2009; Marguerit et al., 2009; Blasi et al., 2011; Moreira et al., 2011; Schwander et al., 2011; Venuti et al., 2013; Ochßner et al., 2016) in order to access the location of grapevine subtilases within these loci.

### Phylogenetic analysis

Two phylogenetic analyses were carried out. The first one with all the identified grapevine subtilase amino acid sequences (97) and a second one combining these amino acid sequences with 56 SBTs amino acid sequences from *A. thaliana* (Rautengarten et al., 2005) and 14 from *Solanum lycopersicum* described in Meichtry et al. (1999).

Protein sequences were obtained from the NCBI database and aligned using the MAFFT software with the L-INS-i option (version 7, Katoh and Standley, 2013; http://mafft.cbrc.jp/alignment/software/), gaps were manually checked and edited in BioEdit v. 7.2.5 (Hall, 1999). A maximum likelihood (ML) phylogenetic analysis was performed with RAxML-HPC v.8, on CIPRES Science Gateway (Miller et al., 2010; https://www.phylo.org), with the following parameters: protein substitution model PROTCAT; protein substitution model + BLOSUM62; bootstrap 1000 iterations with rapid bootstrap analysis (−f a). Multiple alignment uncertainty was scored with ZORRO, assigning a confidence score for each column and applied as (−a) option in RAxML-HPC v.8. Both trees were viewed on FIGTree (http://tree.bio.ed.ac.uk/software/figtree/) and edited on Inkscape (http://www.inkscape.org/).

### Selection of grapevine subtilase sequences putatively involved in pathogen resistance

Previous studies in plants associated some subtilases with the defense response to pathogen attack, like the subtilase SBT3.3 in *A. thaliana* (Ramírez et al., 2013), the P69 in *S. lycopersicum* (Tornero et al., 1996; Jordá et al., 1999) and the cucumisin in grapevine (Figueiredo et al., 2008, 2012). The subtilase genes from *V. vinifera* were blasted against the *A. thaliana* genome (TAIR database, https://www.arabidopsis.org/) and the tomato genome (SolGenomics database, https://solgenomics.net/) to retrieve the grapevine sequences presenting higher sequence similarity to *A. thaliana* SBT3.3 and tomato P69 genes. SolGenomics results were corroborated in NCBI BLAST tool, restricting to *S. lycopersicum* organism, and was assumed the NCBI accession for further studies. Moreover, subtilase sequences with a chromosomal location near the RPV locus on grapevine genome were also selected for further studies. Multiple alignment of the grapevine subtilases selected as putatively involved in plant resistance was performed in DNASTAR software (version 13, Burland, 1999; http://www.dnastar.com/).

### Gycosylation and protein-protein interaction network predictions

Protein glycosylation prediction was done using the NetNGlyc online server (version 1.0, Gupta and Brunak, 2002; http://www.cbs.dtu.dk/services/NetNGlyc/).

The protein interaction network of the selected subtilases was obtained (STRING, version 10.0, Szklarczyk et al., 2014; http://string-db.org/). The gene accessions for all proteins that interact with the selected grapevine subtilases were queried at the NCBI database. The gene ontology (GO) terms for all the interacting proteins were also obtained with the Blast2GO tool (version 3.3, Conesa et al., 2005; https://www.blast2go.com/).

### Experimental design for expression analysis: plant material and inoculation experiments

Two expression analysis experiments were conducted: (1) using two grapevine cultivars (resistant and susceptible) inoculated with *P. viticola* to access subtilase expression during inoculation; (2) using several grapevine accessions with different degrees of resistance toward *P. viticola* to access subtilase constitutive expression.

For the first analysis, two *Vitis vinifera* cultivars were selected to access subtilase expression during interaction with *P. viticola*. The cultivar Regent, bread by multiple introgressions from resistant wild genotypes (Welter et al., 2007), presenting a high degree of resistance to downy and powdery mildews (Anonymous, 2000), and Trincadeira, a Portuguese traditional grapevine cultivar widely used for quality wine production and highly sensitive to *P. viticola* (Figueiredo et al., 2008). Both cultivars were propagated under identical greenhouse conditions, briefly grapevine wood cuttings were grown in 12 cm diameter pots in Fruhstorfer Erde (soil) Type P for 10 weeks, under natural day/night rhythm with temperatures ranging between 5 and 28°C, according to Figueiredo et al. (2012). For plant inoculation, *P. viticola* sporangia were collected after an overnight incubation of symptomatic leaves from greenhouse infected plants in a moist chamber at room temperature. Sporangia were carefully recovered by brushing, dried, stored at −25°C and checked for their vitality by microscopy as in Kortekamp et al. (2008). A suspension containing 10^4^ sporangia ml^−1^ was used to spray the abaxial leaf surface in order to challenge the plants. Mock inoculations with water were also made. After inoculation, plants were kept in a moist chamber (100% humidity) for 8 h and then under greenhouse conditions at 25°C during the inoculation time course. The third to fifth fully expanded leaves beneath the shoot apex were harvested at 6, 12, and 24 hpi, immediately frozen in liquid nitrogen and stored at −80°C. For each genotype and condition (inoculated and mock inoculated), three independent biological replicates were collected, being each biological replicate a pool of three leaves from three different plants.

For the second analysis, to access if subtilases are constitutively expressed, young leaves from several resistant (*V. labrusca, V. rupestris, V. rotundifolia, V. riparia, and V. candicans*) and tolerant (*V. sylvestris*) *Vitis* species and *V. vinifera* cultivars [Regent (resistant) and Trincadeira (susceptible)] were harvested from five different plants (per biological replicate), at the Portuguese Grapevine Germplasm Bank at INIA—Estação Vitivinícola Nacional (Dois Portos), (Supplementary Data 1). Leaves were immediately frozen in liquid nitrogen and stored at −80°C. Three biological replicates were collected from each accession.

### RNA extraction and cDNA synthesis

Total RNA was isolated from frozen leaves with the Spectrum™ Plant Total RNA Kit (Sigma-Aldrich, USA), according to manufacturer's instructions. Residual genomic DNA was digested with DNase I (On-Column DNase I Digestion Set, Sigma-Aldrich, USA). RNA purity and concentration were measured at 260/280 nm using a spectrophotometer (NanoDrop-1000, Thermo Scientific) while RNA integrity was verified by agarose gel electrophoresis (1.2% agarose in TBE buffer). Genomic DNA (gDNA) contamination was checked by qPCR analysis of a target on the crude RNA (Vandesompele et al., 2002). Complementary DNA (cDNA) was synthesized from 2.5 μg of total RNA using RevertAid®H Minus Reverse Transcriptase (Fermentas, Ontario, Canada) anchored with Oligo(dT)_23_ primer (Fermentas, Ontario, Canada), according to manufacturer's instructions.

### Quantitative real time PCR

Quantitative RT-PCR (qPCR) experiments were carried out using Maxima™ SYBR Green qPCR Master Mix (2×) kit (Fermentas, Ontario, Canada) in a StepOne™ Real-Time PCR system (Applied Biosystems, Sourceforge, USA). A final concentration of 2.5 mM MgCl_2_ and 0.2 μM of each primer were used in 25 μL volume reactions, together with 4 μL of cDNA as template. Primer sequences and reaction details are provided in Supplementary Data 2.

Thermal cycling for all genes started with a denaturation step at 95°C for 10 min followed by 40 cycles of denaturation at 95°C for 15 s and annealing (Supplementary Data 2) for 30 s. Each set of reactions included a control without cDNA template. Dissociation curves were used to analyse non-specific PCR products. Three biological replicates and two technical replicates were used for each sample. Gene expression (fold change) was calculated by the Hellemans et al. (2007). The reference genes used for the normalization were the previously described in Monteiro et al. (2013). Statistical significance (*p* < 0.05) of gene expression was determined by the Mann–Whitney U test using IBM® SPSS® Statistics version 23.0 software (SPSS Inc., USA).

## Results and discussion

### Identification and characterization of subtilisin-like serine protease genes in grapevine

The first characterization of the grapevine subtilase family was made by Cao and co-workers in 2014, where 80 subtilase genes were identified (Cao et al., 2014). Subtilase search was restricted to the subtilase conserved PA domain, although subtilases are usually characterized by three conserved domains (PA, S8 peptidase and I9 inhibitor). In parallel, the grapevine genome was re-annotated (Vitulo et al., 2014), nine of the previously identified genes were completely removed from the databases and eight were re-annotated. Thus, we have performed a new characterization of this family in grapevine using the subtilase PA, S8 peptidase and I9 inhibitor domains as query in the new grapevine genome annotation version. Eighty-two *V. vinifera* subtilase genes were identified, from which it is predicted to obtain 97 subtilase proteins (Supplementary Data 3). This search resulted in the introduction of 17 new subtilase genes and the re-annotation of 8 from the subtilase genes previously identified (Cao et al., 2014). The number of genes members found in *V. vinifera* subtilase family is similar to the one from *P. trichocarpa* (90 subtilase genes; Schaller et al., 2012) and potato (82 subtilase genes; Norero et al., 2016) and higher than those reported in other plant species, like Arabidopsis or tomato, which were detected 56 and 15 subtilase genes, respectively (Meichtry et al., 1999; Rautengarten et al., 2005).

**Figure 1:**
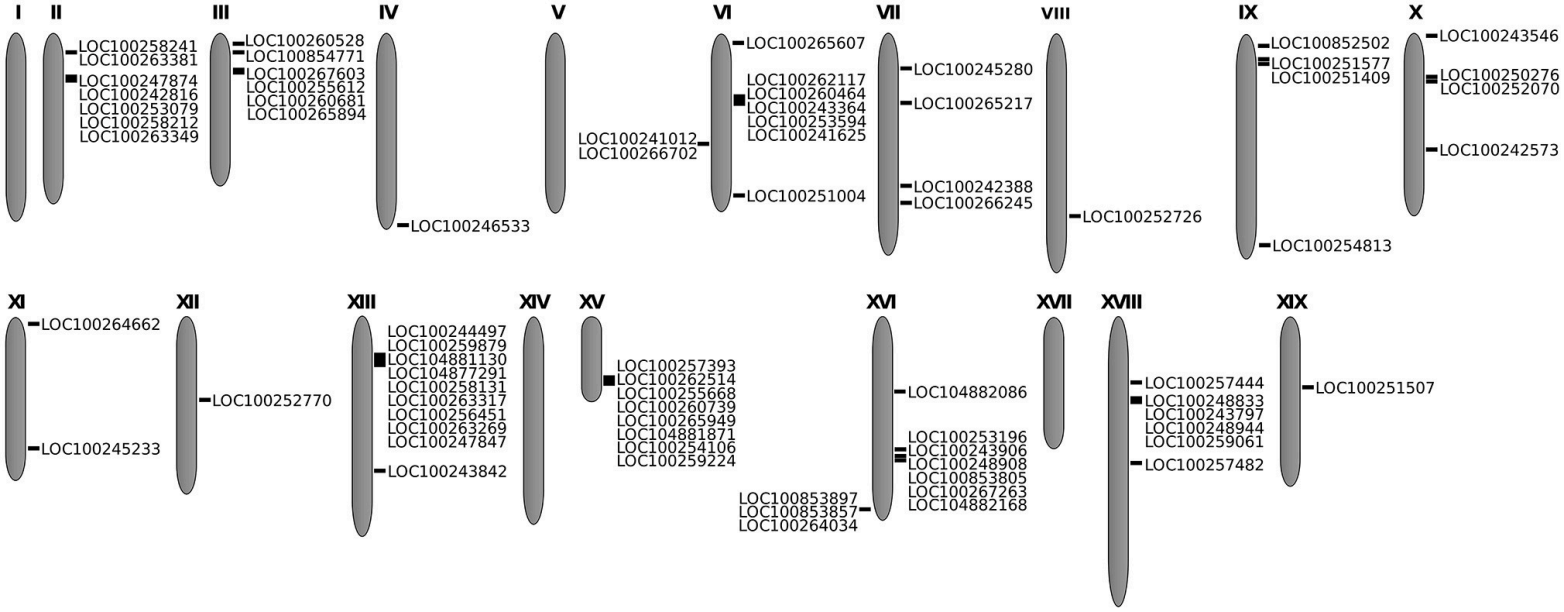
**Location of the 82 subtilase genes in the grapevine chromosomes**. In each illustrated chromosome, subtilase gene accession is shown.

The 82 identified genes identified were mapped in *V. vinifera* chromosomes. These genes were unevenly distributed among 15 of the 19 grapevine chromosomes (Figure 1). No subtilase genes were detected on chromosomes 1, 5, 14, and 17, and the specific location of 8 of the 82 subtilase genes is still unknown. The majority of the subtilase genes were located on chromosomes 6, 13, and 16, with 9 genes in chromosome 6 and 10 in chromosomes 13 and 16 (Figure 1).

### Gene structure analysis

Grapevine STB genes were checked for exon-intron structure. Details of the exon-intron structures are shown in Supplementary Data 3. The number of introns varied between 0 and 18, around 24% of the grapevine subtilase genes are intronless and 5% present a high number of introns (17–18 introns). Intronless subtilase genes have been reported in Arabidopsis and potato (Rautengarten et al., 2005; Norero et al., 2016) being the highest number of introless genes reported in potato (63% of the *St*SBT genes; Norero et al., 2016). Intronless genes can serve as beacons in analyses of gene function and evolution, they have been found in large gene families and related to gene duplications, inheritance from ancient prokaryotes, retroposition or other mechanisms (Yan et al., 2016). In general, most closely related members in the same group shared a similar exon-intron structure.

### Protein structure and domain analysis

Both molecular weight (Mw) and isoelectric point (pI) were predicted for the grapevine subtilase proteins (Supplementary Data 3). *V. vinifera* subtilase proteins have a wide range of molecular weights, between 19 and 164 kDa, slightly higher than the already described for other plant serine proteases (between 19 and 110 kDa; Antão and Malcata, 2005). The majority (60%) present a theoretical molecular weight between 80 and 90 kDa, and only 26% between 60 and 80 kDa as previously described for other plant serine proteases (Antão and Malcata, 2005). The remaining 14% presented a molecular weight lower than 60 kDa or higher than 90 kDa. Grapevine subtilases have a theoretical pI between 4.69 and 9.57. This pI range is comparable to other subtilase proteins, like, for example the STB3.3 (AT1G32960.1) from *A. thaliana* and P69C (CAA76726) from tomato with a theoretical pI of 6.27 and 5.27, respectively (predicted from protein sequence with Compute pI/Mw tool from ExPASy, http://web.expasy.org/compute_pi/) or the *Cp*SUB1 from papaya with a pI of 8.97 (Othman and Nuraziyan, 2010).

Subtilases are characterized by a multidomain structure comprising a signal peptide, a propeptide, a protease domain (S8) and a protease-associated (PA) domain (Siezen et al., 2007). The protease domain (S8 domain) that defines the subfamily S8A (PF00082), includes the catalytic triad and a protease-associated domain (PA) (PF02225) which is an insertion of about 120–160 amino acids long between the His and Ser active site residues that cause a displacement of the reactive Ser from the catalytic triad to the C-terminal (Siezen and Leunissen, 1997). This domain has been implicated in protein-protein interactions or substrate specificity (Siezen and Leunissen, 1997). All of the 97 grapevine subtilases identified present the S8 domain (Supplementary Data 3) and 6 subtilase sequences have S8 domain duplication (Supplementary Data 3). Ninety of the *VvSBT*s presented the I9 inhibitor domain and the same sequences that contained S8 domain duplication also presented duplication of the I9 domain (Supplementary Data 3). This I9 inhibitor domain (PF05922), also known as pro-domain N terminus or propeptide, is involved after removal, on the pro-enzyme activation, working as a molecular chaperone in the folding of the mature peptidase. Thus, the I9 inhibitor prevents the access of the substrate to the active site and activates the peptidase when it is removed either by autocatalytic cleavage or by interaction with a secondary peptidase (Siezen, 1996). The PA domain was detected in all of the subtilase sequences, but only in 46 sequences the E-value was considered significant (*E* ≤ −5). On the other 39 sequences the presence of the PA domain presents low E values or is listed as unintegrated signature. However, the shift on the reactive Ser from the catalytic triad to the C-terminal is present in all of the grapevine subtilase sequences, which may suggest sequence divergence for this domain.

Not all the grapevine subtilases exhibited the three domains simultaneously. Despite being conserved in plant subtilases, the simultaneous presence of I9 inhibitor, S8 peptidase, and PA domain is not obligatory requisite. Moreover, it is yet to be confirmed if the simultaneous presence or not of this set of domains has some effects in subtilase functions. An example of the non-simultaneous existence of the three conserved domains is the P69C subtilase from tomato (Tornero et al., 1997).

The presence of subtilases with domain repeats can be a result of the evolution and a way to improve the subtilase features and its functions. Gene duplication and mutation processes in biological evolution have been largely recognized since the 1930s (Bridges, 1936; Brown and Doolittle, 1995; Zhang, 2003). Gene duplication may result in domain repeats in protein structure. These repeats have a rich variety of functional properties involving protein-protein interactions as well as binding to other molecules like DNA or RNA. Furthermore, long tandem of repeats can play an important role in the folding of three dimensional structures of multi-domain proteins. Structural studies in proteomics have shown that the abundance of domain repeats in organisms of higher complexity is highly correlated with domain families involved in complex-assembly, cell-adhesion, and signaling processes (Han et al., 2007).

Six *Vv*SBT presented an additional domain, the fibronectin (Fn) III-like domain (PF06280), (Supplementary Data 3). This domain of unknown function is required by some plant subtilases for their activity (Rawlings and Salvesen, 2013). The SBT3.3 subtilase structure from *A. thaliana* showed the presence of the three conserved domains and also a fibronectin (Fn) III-like domain (Rose et al., 2010).

### Subcellular location prediction

Predictions of the subcellular location of a gene product can provide additional information for its functional involvement. Different subcellular locations of plant subtilases have been found to correlate with their different physiological functions (Rautengarten et al., 2005; Cao et al., 2014). For example, the *Cp*SUB1 subtilase from papaya is secreted to extracellular space, where it plays a role in the early stage of fruit development and ripening by degrading cell wall matrix (Othman and Nuraziyan, 2010). Rice subtilase RSP1 is only present in the reproductive organ and absent in leaves, roots, embryos, or rice panicles (Yoshida and Kuboyama, 2001). This suggests that the role for each plant subtilase is related to its location event in spite of analogous structural features (Othman and Nuraziyan, 2010). Sixty *Vv*SBTs possess signal sequences for targeting to the secretory pathway (S), 26 subtilases do not contain any known targeting motif, 8 family members are predicted to be targeted to mitochondria (M) and 3 to the chloroplast (C), (Supplementary Data 4). Hence, grapevine subtilases may have a diversity of functions, most of them probably with roles in the extracellular space or matrix.

### Phylogenetic analysis of grape subtilases

**Figure 2:**
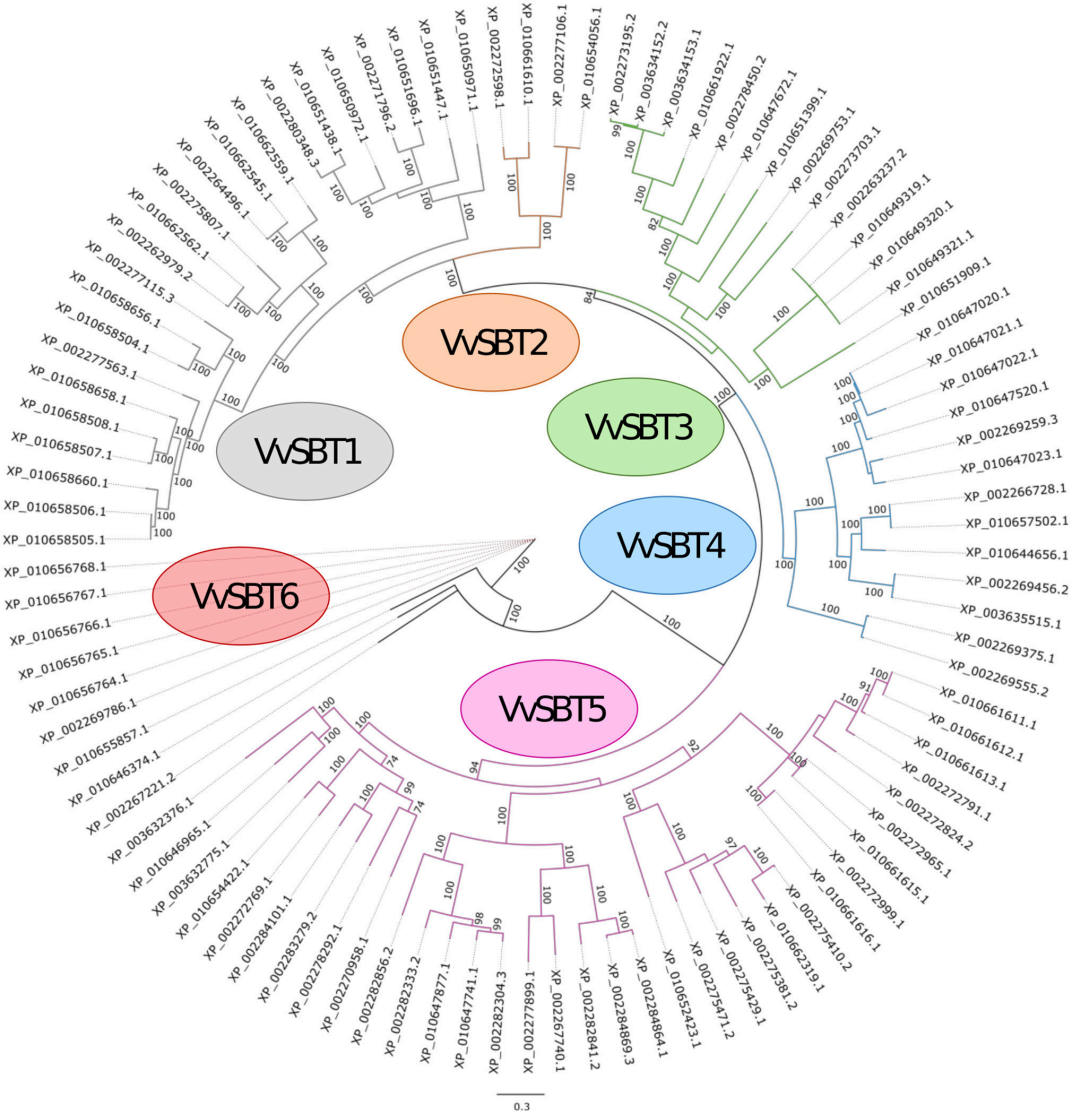
**Maximum likelihood phylogenetic tree of the 97 subtilisin-like serine proteases from ***Vitis vinifera*****. The six groups are shown (*Vv*SBT1–*Vv*SBT6) and three sequences were defined as outgroup (XP_010655857.1, XP_010646374.1, and XP_002267221.2). The numbers above branches show bootstrap values. Scale bar represents the number of estimated changes per branch length.

A phylogenetic analysis of the 97 grapevine subtilase proteins was carried out and the consensus phylogeny obtained is shown in Figure 2. Based on the phylogenetic relationships of the grapevine subtilases proteins found, an outgroup was identified and 6 SBT groups were established and named *Vv*SBT1 to *Vv*SBT6 (Figure 2). *Vv*SBT1 comprise 23 subtilases and include all the subtilases annotated as cucumisin (degradative subtilases from *Cucumis melo)*. *Vv*SBT1 coding genes are unevenly distributed among grapevine chromosomes and present a structure with variable number of introns (Supplementary Data 3). *Vv*SBT2 is the smallest group containing 4 subtilase proteins annotated as xylem serine proteins. All of the coding genes present a 10 intron structure and are distributed between chromosome 8 and 15 (Supplementary Data 3). The *Vv*SBT3 group comprises 14 subtilases proteins, most of them presenting similarity with *A. thaliana* SBT3.3/SBT3.5 proteins. All of the coding genes present more than 9 introns and an uneven chromosomal location (Supplementary Data 3). *Vv*SBT4 includes 13 subtilase proteins, most of them showing similarity to *A. thaliana* SBT5.3/SBT5.4. Within this group subtilase coding genes present between 5-10 introns and for most of the genes the chromosomal location is unknown (Supplementary Data 3). *Vv*SBT5 is the largest group including 34 subtilase proteins that are annotated as subtilisin-like proteins. Within this group, most of the coding genes are intronless or present 1–2 intron. *Vv*SBT6 is comprised by 6 subtilase proteins all containing an additional fibronectin III-like domain, the coding genes are all located in chromosome 11 and present 13 introns (Supplementary Data 3). Rautengarten and co-workers have equally performed a phylogenetic analysis of the predicted 56 *A. thaliana* (*At*SBT) subtilase sequences that showed a division of the subtilases into 6 groups (Rautengarten et al., 2005). In potato, 5 subtilase groups were considered (Norero et al., 2016).

### Phylogenetic analysis of grapevine, tomato, and Arabidopsis subtilases

**Figure 3:**
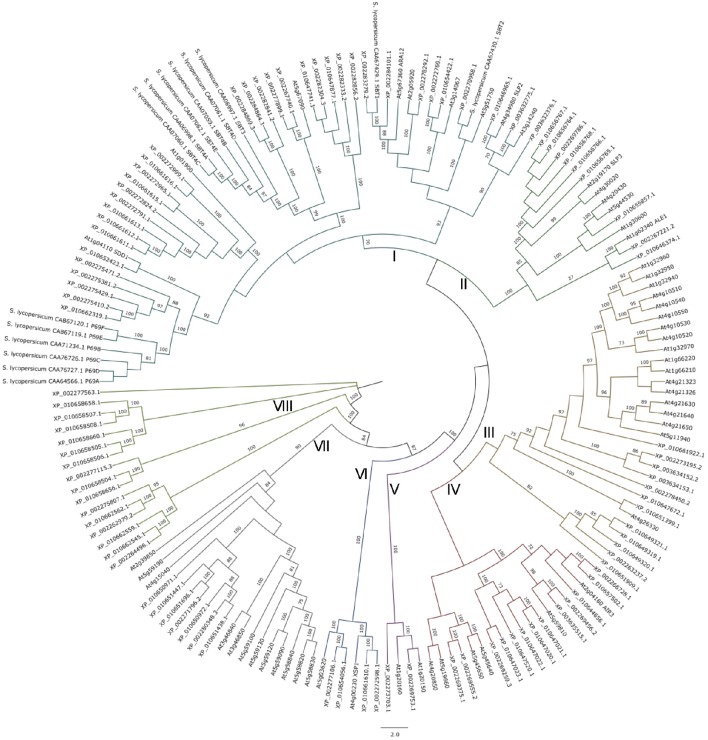
**Maximum likelihood phylogenetic tree of 97 subtilisin-like serine protease proteins from ***Vitis vinifera***, 14 from ***Solanum lycopersicum***, and 56 from ***Arabidopsis thaliana*****. Eight clusters named from I to VIII are shown. The numbers above branches show bootstrap values. Scale bar represents the number of estimated changes per branch length.

Biological functions of plant subtilases remain largely unknown. The phylogenetic analysis of 168 amino acid sequences including grapevine, tomato and Arabidopsis subtilases evidenced 8 clusters named from I to VIII (Figure 3).

Cluster I consists in all the tomato subtilase proteins, and all members from the *At*SBT1 and *Vv*SBT5 groups. In this cluster grapevine subtilase proteins XP_010662319.1, XP_002275410.2, XP_002275429.1, XP_002275381.2, XP_002275471.2 are closely related to tomato P69 genes already described as being associated to biotic stress responses (Granell et al., 1987; Vera and Conejero, 1988; Christ and Mösinger, 1989; Fischer et al., 1989; Vera et al., 1989; Tornero et al., 1997; Jordá et al., 1999, 2000; Meichtry et al., 1999). XP_010652423.1 is the homolog of Arabidopsis SDD1 (stomatal density and distribution 1 protease) involved in the regulation of stomata distribution and density during leaf development (Von Groll et al., 2002) and XP_002284101.1, XP_002283279.2 could be related to Arabidopsis ARA12 that participates in seed germination (Rautengarten et al., 2008).

Cluster II includes the grapevine *Vv*SBT6 group, the three subtilase sequences considered as an outgroup (Figures 2, 3) and the *At*SBT2 group (which included the ALE1 protease necessary for cuticle formation and epidermal differentiation during embryo development in *A. thaliana* (Tanaka et al., 2001).

Cluster III comprises the *At*SBT3 group including the *At*SBT3.3 recently described as being involved in immune priming events in Arabidopsis (Ramírez et al., 2013) and several proteins from the *Vv*SBT3 group (Figure 2).

Cluster IV groups the grapevine *Vv*SBT4 group and the *At*SBT5.3, *At*SBT5.4, *At*SBT5.5, *At*SBT5.6, *At*SBT6.1, and *At*SBT6.2 subtilases. The grapevine XP_010657502.1, member of the *Vv*SBT4 group appears closely related to the *At*SBT5.3 is encoded by the AIR3 involved on lateral root formation (Neuteboom et al., 1999). Another member of *Vv*SBT4, the subtilase XP_002269375.1, is also closely related to Arabidopsis SBT6.1 which is involved in the unfolded protein response through the cleavage of an ER-resident type II membrane protein (bZIP28; Liu et al., 2007; Che et al., 2010; Liu and Howell, 2010a,b).

Cluster V includes two grapevine subtilases from SBT3 group, both named CO(2)-response secreted protease and two *At*SBT subtilases, AtSBT5.1 and 5.2, suggesting that these subtilases may share common functions.

Cluster VI groups the *Vv*SBT2 group and the *At*SBT4.14 and SBT4.15 involved in the xylem differentiation (Zhao et al., 2000).

Cluster VII is defined by the remaining members of the *At*SBT4 group and seven grapevine subtilases from the *Vv*SBT1 group, all presenting the coding genes located in chromosome 6 (Supplementary Data 3).

Clusters VIII includes all of the remaining sequences from the *Vv*SBT1 group, being located in chromosome 16 and 13, respectively. These *Vv*SBT could have evolved separated from Arabidopsis subtilases.

### Selection of subtilases putatively involved in grapevine immunity

The subtilases SBT3.3 from *A. thaliana* and P69 from tomato are by far the most studied and associated to defense responses to pathogen attack (Tornero et al., 1997; Jordá et al., 1999; Ramírez et al., 2013). Tomato P69 subtilases present at least six closely related genes (P69A to P69F; Tornero et al., 1997; Jordá et al., 1999, 2000; Meichtry et al., 1999), but only P69B and P69C were shown to behave as pathogenesis-related (PR) genes being induced by pathogen infection and salicylic acid (Tornero et al., 1997; Jordá et al., 1999). *A. thaliana* SBT3.3 is the only subtilase associated to defense mechanisms, this subtilase gene is embedded in a genomic cluster encompassing three additional subtilases (*SBT3.5, SBT3.4, and SBT3.2*; Ramírez et al., 2013). Also, our previous studies have highlighted the potential involvement of a subtilase (XM_010660203.1) in the defense response of grapevine against the biotrophic oomycete *P. viticola* (Figueiredo et al., 2008, 2012; Monteiro et al., 2013). Along with this subtilase, we have selected several grapevine subtilases putatively involved in the defense against *P. viticola* for expression studies (Table 1) based on their sequence identity (higher than 50%) with *At*SBT3.3 and the tomato P69C (XM_002273159.3, XM_002275345.2, XM_002275374.2, XM_002275393.2, XM_002275435.2, XM_002278414.3, XM_003634104.2, XM_003634105.2, XM_010649370.1, XM_010663620.1).

**Table 1:** **Grapevine subtilase proteins presenting sequence similarity with SBT3.3, SBT3.5, and P69C from ***Arabidopsis thaliana*** and ***Solanum lycopersicum*****.

**Nucleotide**	***S. lycopersicum***	**NCBI ID**	**Identity (%)**	***E*-value**	***A. thaliana***	**TAIR ID**	**Identity (%)**	***E*-value**
XM_002263201.3	SBT3.5	XP_004233282.1	75	0	SBT3.5	AT1G32940.1	57	e-148
XM_002267185.3	SBT3.5	XP_004235142.1	66	0				
XM_002269717.2	SBT3.5	XP_004233282.1	43	e-177				
XM_002269750.2	SBT3.5	XP_004235142.1	41	0				
XM_002270922.2	P69C	CAA76726.1	44	e-146				
XM_002271760.2	SBT3.5	XP_004233282.1	41	e-160				
XM_002272562.3	SBT3.5	XP_004233282.1	41	e-167				
XM_002272733.2	P69C	CAA76726.1	46	e-166				
XM_002272755.2	P69C	CAA76726.1	44	e-159				
XM_002272788.3	P69C	CAA76726.1	44	e-176				
XM_002272929.2	P69C	CAA76726.1	46	e-173				
XM_002272963.2	P69C	CAA76726.1	46	e-159				
XM_002273159.3^*^	SBT3.3	XP_010318060.1	59	0	SBT3.3	AT1G32940.1	70	0
XM_002273667.2	SBT3.5	XP_004233282.1	44	e-176				
XM_002275345.2^*^	P69C	CAA76726.1	59	0				
XM_002275374.2^*^	P69C	CAA76726.1	59	0				
XM_002275393.2^*^	P69C	CAA76726.1	57	0				
XM_002275435.2^*^	P69C	CAA76726.1	59	0				
XM_002277079.3	SBT3.5	XP_004233282.1	44	e-156				
XM_002278256.2	P69C	CAA76726.1	46	e-158				
XM_002278414.3^*^	SBT3.3	XP_010318060.1	53	0	SBT3.3	AT1G32960.1	66	0
XM_002280312.3	SBT3.5	XP_004233282.1	41	e-154				
XM_002283243.3	P69C	CAA76726.1	47	e-168				
XM_002284065.3	P69C	CAA76726.1	45	e-162				
XM_003632328.2	P69C	CAA76726.1	46	e-171				
XM_003632727.2	P69C	CAA76726.1	43	e-154				
XM_003634104.2^*^	SBT3.3	XP_010318060.1	59	0	SBT3.3	AT1G32960.1	69	0
XM_003634105.2^*^	SBT3.3	XP_010318060.1	59	0	SBT3.3	AT1G32960.1	70	0
XM_010648663.1	P69C	CAA76726.1	44	e-146				
XM_010649370.1^*^	SBT3.3	XP_010318060.1	50	0	SBT3.5	AT1G32940.1	58	e-161
XM_010651017.1	SBT3.5	XP_004233282.1	76	0	SBT3.5	AT1G32940.1	57	e-146
XM_010651018.1	SBT3.5	XP_004233282.1	77	0	SBT3.5	AT1G32940.1	57	e-146
XM_010651019.1	SBT3.5	XP_004233282.1	75	0	SBT3.5	AT1G32940.1	57	e-146
XM_010652670.1	SBT3.5	XP_004233282.1	42	e-159				
XM_010653097.1	SBT3.3	XP_010318060.1	44	0	SBT3.5	AT1G32940.1	58	e-157
XM_010653136.1	SBT3.5	XP_004233282.1	42	e-151				
XM_010653394.1	SBT3.5	XP_004233282.1	42	e-164				
XM_010653607.1	SBT3.5	XP_004233282.1	46	0				
XM_010654121.1	P69C	CAA76726.1	45	e-163				
XM_010656120.1	P69C	CAA76726.1	46	e-171				
XM_010657555.1	SBT3.5	XP_004235142.1	43	0				
XM_010658462.1	SBT3.5	XP_004235142.1	41	0				
XM_010658463.1	SBT3.5	XP_004235142.1	41	0				
XM_010658464.1	SBT3.5	XP_004235142.1	41	0				
XM_010658465.1	SBT3.5	XP_004235142.1	41	0				
XM_010658466.1	SBT3.5	XP_004235142.1	41	0				
XM_010660205.1	SBT3.5	XP_004233282.1	41	e-157				
XM_010663308.1	SBT3.5	XP_004233282.1	39	e-154				
XM_010663309.1	P69C	CAA76726.1	43	e-156				
XM_010663310.1	P69C	CAA76726.1	43	e-156				
XM_010663311.1	P69C	CAA76726.1	44	e-156				
XM_010663313.1	P69C	CAA76726.1	46	e-174				
XM_010663314.1	P69C	CAA76726.1	46	e-160				
XM_010663620.1^*^	SBT3.3	XP_010318060.1	59	0	SBT3.3	AT1G32960.1	78	0

Gene identification, sequence identity (%) and E-value are indicated. Asterisk (

* *) indicate subtilases selected for further studies*.

Several grapevine chromosomal locus associated with the resistance to *P. viticola* (named “*Resistance to P. viticola”*—RPV) have been identified (Merdinoglu et al., 2003; Fischer et al., 2004; Wiedemann-Merdinoglu et al., 2006; Welter et al., 2007; Bellin et al., 2009; Marguerit et al., 2009; Blasi et al., 2011; Moreira et al., 2011; Schwander et al., 2011; Venuti et al., 2013; Ochßner et al., 2016). Chromosomal location of the previously identified grapevine subtilase genes was compared with the location of the known RPV's in *V. vinifera* chromosomes (Table 2). Three subtilase genes were selected: XM_002277863.3 and XM_002284065.3 located at 16.7 and 15.7 Mb (within the *Rpv9* in chromosome 7) and XM_010659200.1 located at 10.2 Mb between two RPV's, (*Rpv1* situated at 10.3 Mb and *Rpv13* placed at 10.0 Mb in chromosome 12; Table 2).

**Table 2:** **Traits and allelles associated with ***Vitis*** resistance to ***Plasmopara viticola*****.

**Grapevine Gene locus**	**Nucleotide**	**Position (Mb)**	**Chromosome**	**RPV**	**Associated marker**	**Chromosome position (Mb)**	**References**
LOC100246533	XM_002263201.3	23.8	4	*Rpv4*	VMC7h3	4.7	Welter et al., 2007
	XM_010651017.1				VMCNg2e1	5.2	
	XM_010651018.1						
	XM_010651019.1						
LOC100266245	XM_002277863.3^*^	16.7	7	*Rpv7*	UDV-097	11.4	Bellin et al., 2009; Moreira et al., 2011
LOC100242388	XM_002284065.3^*^	15.7		*Rpv9*	CCoAOMT	16.6	
LOC100245280	XM_003632328.2	3.5					
LOC100265217	XM_010654121.1	7.5					
LOC100254813	XM_002267704.2	21.9	9	*Rpv5*	VVIo52b	4.0	Marguerit et al., 2009; Schwander et al., 2011
LOC100251409	XM_002272733.2	0.8		*Rpv10*	GF09-46	3.7	
LOC100852502	XM_003632727.2	0.3					
LOC100251577	XM_010656120.1	0.8					
LOC100252770	XM_002266692.2	10.2	12	*Rpv1*	VVIb32	10.3	Merdinoglu et al., 2003; Marguerit et al., 2009; Moreira et al., 2011
	XM_010659200.1^*^			*Rpv6*	VMC8G9	20.4	
							
				*Rpv13*	VMC1G3.2	10.0	
LOC100257482	XM_002269717.2	12.9	18	*Rpv3*	UDV-112	24.9	Welter et al., 2007; Bellin et al., 2009
						26.9	
LOC100259061	XM_002282805.2	8.7			UDV-305		
LOC100248833	XM_002282820.2	8.6			VMC7f2		
LOC100257444	XM_002283243.3	7.9					
LOC100248944	XM_002284828.3	8.7					
LOC100243797	XM_002284833.3	8.6					

Comparison between chromosomal location of grapevine subtilase genes and chromosomal location of “Resistance to Plasmopara viticola—RPV” loci. Asterisk (

* *) indicate subtilases selected for further studies*.

The 14 selected grapevine subtilases potentially linked to *V. vinifera* immunity were further analyzed. A prediction of glycosylated sites was performed in the selected grapevine proteins (Table 3) as it has been shown that glycosylated plant subtilases are secreted to plant extracellular matrix (ECM; Bykova et al., 2006; Cedzich et al., 2009). Since the ECM is where the first host-pathogen interaction, recognition and signaling events take place (Dixon and Lamb, 1990), the accumulation of subtilases in plant ECM may account for an important role during pathogenesis. The most important protein glycosylation form is *N*-linked, formed by the covalent attachment of asparagine-linked carbohydrates to the protein (Gupta and Brunak, 2002; Bykova et al., 2006). Protein *N*-glycosylation was previously described in subtilases P69B from tomato (Bykova et al., 2006). From the 14 protein sequences analyzed, only two may not contain a signal peptide (XM_002275345.2, XM_010659200.1), and thus may not be glycosylated *in vivo*, even though they contain potential motifs. The remaining 12 proteins seem to contain a signal peptide and *N*-glycosylation was predicted in several Asp residues (Table 3).

**Table 3:** **Signal peptide, N-glycosylation, and subcellular location prediction for the subtilases putatively involved in grapevine immunity**.

**Nucleotide**	**Sequence identification (NCBI)**	***Vv*SBT group**	**Chromosomal location**	**selection**	**Signal peptide prediction, signalP 4.1 (position)**	***N*-glycosylation prediction (Asn-X-Ser/Thr), NetNGlyc 1.0 Server (position and sequence)**	**Subcellular location prediction**
XM_002273159.3	Subtilisin-like protease SBT3.5	*Vv*SBT3	16	Similarity to *At*SBT3.3	YES (1–27)	YES	Extracellular region
						(184 **N**AT)	
						(212 **N**TT)	
						(373 **N**RT)	
						(385 **N**HT)	
						(412 **N**DT)	
						(646 **N**NS)	
						(682 **N**ST)	
						(689 **N**VT)	
						(697 **N**ST)	
XM_002275345.2	Subtilisin-like protease SDD1	*Vv*SBT5	2	Similarity to P69C	NO	YES ^*^	Apoplast; Plant-type cell wall
						(15 **N**GT)	
						(259 **N**CS)	
						(314 **N**VS)	
						(951 **N**DT)	
XM_002275374.2	Subtilisin-like protease SDD1	*Vv*SBT5	2	Similarity to P69C	YES (1–31)	YES	Apoplast; Plant-type cell wall
						(137 **N**RS)	
						(186 **N**GT)	
						(366 **N**FS)	
						(382 **N**QT)	
						(526 **N**VT)	
						(644 **N**CS)	
XM_002275393.2	Subtilisin-like protease	*Vv*SBT5	2	Similarity to P69C	YES (1–20)	YES	Apoplast; Plant-type cell wall
						(306 **N**ST)	
						(510 **N**RT)	
						(615 **N**DT)	
XM_002275435.2	Uncharacterized protein LOC100242816	*Vv*SBT5	2	Similarity to P69C	YES (1–31)	YES	Apoplast; Plant-type cell wall
						(254 **N**GT)	
						(338 **N**GS)	
						(398 **N**AS)	
						(542 **N**DT)	
						(656 **N**RT)	
						(699 **N**SS)	
						(1053 **N**TT)	
						(1196 **N**ST)	
XM_002277863.3	Subtilisin-like protease	*Vv*SBT5	7	*Rpv9*	YES (1–23)	YES	Apoplast; Plant-type cell wall
						(184 **N**FT)	
						(216 **N**SS)	
						(247 **N**GT)	
						(298 **N**NS)	
						(421 **N**AT)	
						(460 **N**KS)	
						(588 **N**DT)	
						(614 **N**AT)	
						(687 **N**YT)	
						(719 **N**LT)	
XM_002278414.3	Subtilisin-like protease SBT3.3	*Vv*SBT3	2	Similarity to *At*SBT3.3	YES (1–35)	YES	Extracellular region
						(192 **N**ST)	
						(219 **N**IT)	
						(424 **N**AT)	
						(642 **N**IS)	
						(734 **N**LT)	
XM_002284065.3	Subtilisin-like protease	*Vv*SBT5	7	*Rpv9*	YES (1–24)	YES	Extracellular region
						(172 **N**FT)	
						(378 **N**AS)	
						(633 **N**YS)	
XM_003634104.2	Subtilisin-like protease SBT3.5	*Vv*SBT3	16	Similarity to *At*SBT3.3	YES (1–27)	YES	
						(184 NAT)	
						(212 NTT)	
						(373 NRT)	
						(385 NHT)	
						(412 NDT)	
						(646 NNS)	
						(682 NST)	
						(689 NVT)	
						(697 NST)	
XM_003634105.2	Subtilisin-like protease SBT3.5	*Vv*SBT3	16	Similarity to *At*SBT3.3	YES (1–27)	YES	Extracellular region
						(184 **N**AT)	
						(212 **N**TT)	
						(373 **N**RT)	
						(385 **N**LT)	
						(412 **N**DT)	
						(646 **N**NS)	
						(682 **N**ST)	
						(689 **N**VT)	
						(697 **N**ST)	
						(722 **N**ST)	
XM_010649370.1	Subtilisin-like protease SBT3.5	*Vv*SBT3	3	Similarity to *At*SBT3.3	YES (1–27)	YES	Extracellular region
						(179 **N**RS)	
						(362 **N**QT)	
						(407 **N**AT)	
						(651 **N**TT)	
XM_010659200.1	Subtilisin-like protease SBT5.3 isoform X2	*Vv*SBT4	12	*Located within Rpv1 + Rpv13*	NO	YES ^*^	
						(60 **N**SS)	
						(95 **N**GT)	
						(155 **N**DS)	
						(245 **N**AS)	
						(318 **N**FT)	
						(330 **N**ST)	
						(455 **N**AS)	
						(500 **N**QT)	
XM_010660203.1	Cucumisin isoform X1	*Vv*SBT1	13	(Figueiredo et al., 2008, 2012; Monteiro et al., 2013)	YES (1–29)	YES	Extracellular region
						(180 **N**FT)	
						(387 **N**RS)	
						(460 **N**ST)	
						(640 **N**GT)	
						(666 **N**RT)	
XM_010663620.1	Subtilisin-like protease SBT3.5	*Vv*SBT3	2	Similarity to *At*SBT3.3	YES (1–36)	YES	Extracellular region
						(188 **N**ST)	
						(216 **N**TS)	
						(247 **N**VS)	
						(377 **N**KT)	
						(925 **N**TT)	
						(956 **N**AS)	
						(1086 **N**VT)	

Asterisk (

* *) indicates sequences that may not contain a signal peptide*.

The protein–protein interaction network for the selected subtilases putatively involved in grapevine immunity was performed. By understanding the protein environment where these proteins are likely involved, it is feasible to obtain relevant information about their function and the biological processes. For this analysis, the STRING database was used (Szklarczyk et al., 2014). The top 50 proteins that interact with the 14 grapevine subtilases were analyzed individually in UniProt to access the biological processes to which they are associated. Five of these proteins were predicted to interact with all of the selected grapevine subtilases and are involved in biological processes associated to defense responses, namely fatty acid beta-oxidation, protein kinase activity, ER-associated ubiquitin-dependent protein catabolic process, defense response and protein serine/threonine kinase activity (Supplementary Data 5). Lipid peroxidation and lipid-associated signaling have been recently associated to grapevine resistance to *P. viticola* (Figueiredo et al., 2015; Guerreiro et al., 2016). Also protein kinases are known to regulate the majority of cellular pathways, especially those involved in signal transduction (Dhanasekaran and Premkumar Reddy, 1998). This protein-protein interaction network result reinforces the hypothesis that these 14 subtilases may have some involvement in the grapevine immunity.

### Expression analysis

Expression profiles of the selected grapevine subtilases were first analyzed in two *V. vinifera* cultivars, Regent and Trincadeira (resistant and susceptible to *P. viticola*, respectively) at 6, 12 and 24 h after inoculation (hpi) with *P. viticola*. Early inoculation time points were chosen to access the signaling events during pathogen recognition: between 6 and 12 hpi stomatal penetration and development of stomatal vesicles with primary hyphae occurs and at 24 hpi elongated hyphae invade the intercellular space of the mesophyll progressing to the branching stage in susceptible plants and stopping the development in resistant plants (Kortekamp and Zyprian, 2003; Unger et al., 2007).

**Figure 4:**
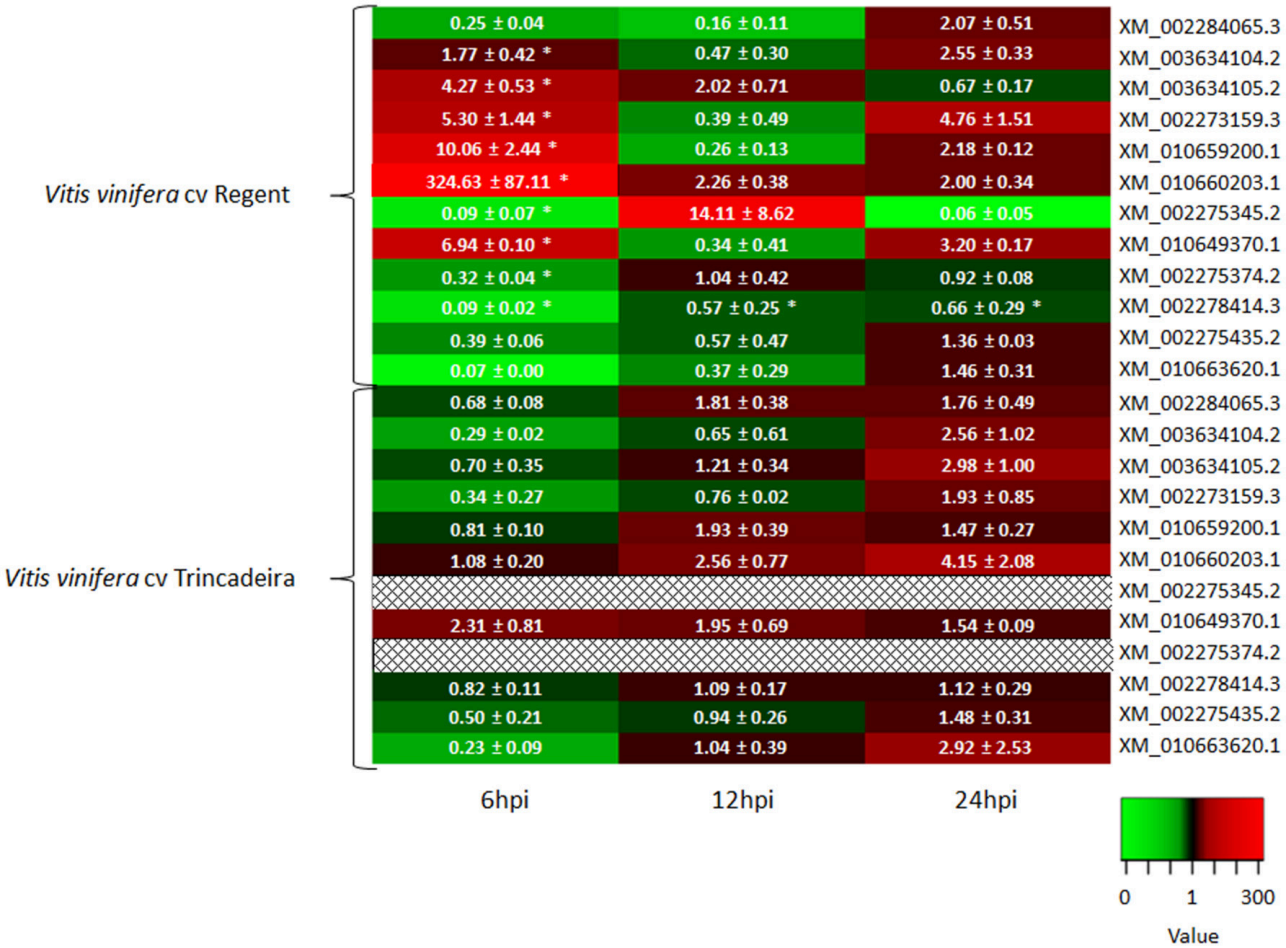
**Heatmap of the 14 grapevine subtilase expression in ***V. vinifera*** cv Regent and ***V. vinifera*** cv Trincadeira at 6, 12, and 24 hpi with ***P. viticola*****. Each column indicates a time-point (6, 12, and 24 hpi) and each row represents a subtilase gene in the resistant grapevine genotype (Regent) or in the susceptible grapevine genotype (Trincadeira) and was colored according to the log2 ratio of expression. Green indicates lower expression, red indicates higher expression, black indicates no expression (see the color scale) and 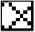 indicates no amplification. Asterisks (^*^) represent significant difference (*p* ≤ 0.05) between target and control samples (Mann–Whitney *U*-test; SPSS Inc., USA, V20).

Of the 14 grapevine subtilases analyzed, two presented no amplification (XM_002275393.2: sequence similarity with P69C and XM_002277863.3: located in *rpv9*) in both cultivars and were retrieve from our study. Three subtilases that present either sequence homology with *At*SBT3.3 (XM_003634104.2 and XM_002273159.3) or are located in the *rpv* loci (XM_010659200.1) presented the same expression pattern during inoculation time-course in the resistant cultivar Regent, being up-regulated at 6 hpi, decreasing their expression at 12 hpi and increasing again at 24 hpi (Figure 4). In the susceptible genotype, these subtilases were more expressed at 24 hpi (Figure 4). Also, the subtilase XM_003634105.2 (presenting sequence homology with *At*SBT3.3) showed an up-regulation at the early time-points analyzed (6 and 12 hpi) in the resistance cultivar Regent, being down-regulated at 24 hpi. In the susceptible cultivar Trincadeira, this gene was down-regulated at 6 hpi and increased its expression from 12 hpi (Figure 4). Accordingly, in *Vitis pseudoreticulata* leaves infected with the biotrophic ascomycete *Erysiphe ne*cator (Schw.) Burr., presented an up-regulation of all these subtilase genes after infection (Weng et al., 2014).

The grapevine subtilase gene XM_010649370.1, showing homology with *At*SBT3.3, exhibited an increase of expression at both 6 and 24 hpi, being down-regulated at 12 hpi, in the resistant cultivar, while in the susceptible cultivar this gene is over-expressed during all the inoculation time-course (Figure 4). The expression of this subtilase has been previously analyzed in different grapevine tissues and abiotic stimuli (Cao et al., 2014), exhibiting a constitutive high-level of expression in roots, leaves, and stem and the expression was supressed in abiotic (salt, heat, cold, and drought) stress conditions (Cao et al., 2014), which may suggest that it could have a participation in response to biotic stimulus instead. Recently, a *Gossypium babardense* subtilase gene *GbSBT1*, that show a high sequence homology with the grapevine subtilase XM_010649370.1, was associated with the defense response to extracellular stimulations like *Verticillium dahlia* infection, the cause of the Verticillium wilt disease. The corresponding GbSBT1 protein showed to be mainly localized at the cell membrane and moves to the cytoplasm following treatment with jasmonic acid and ethylene, which supports the hypothesis of some grapevine subtilases are located near the place where occurs the plant-pathogen interaction. Moreover, Duan and co-workers, observed a reduction of the tolerance of a cotton resistant genotype, when the *GbSBT1* gene was silenced, and an activation of the expression levels of defense-related genes (Duan et al., 2016).

Two subtilases presenting sequence homology with *At*SBT3.3 (XM_010663620.1) and located at the *rpv9* (XM_002284065.3) showed a similar expression pattern, being up-regulated at the later time-point analyzed (24 hpi) in the resistant cultivar Regent, however in the susceptible cultivar Trincadeira both genes have an earlier and higher expression starting at 12 hpi. The expression of the subtilase XM_002284065.3 was also analyzed by Cao and co-workers and an increase of expression during abiotic stress was shown (Cao et al., 2014). Both results suggest an involvement of this subtilase in response to abiotic and biotic environmental stimulus.

The expression of XM_002278414.3 (presenting homology with *At*SBT3.3) and XM_002275435.2 (presenting homology with tomato P69C) was either not altered or down-regulated during inoculation time-course in both cultivars (Figure 4). Accordingly this subtilase presented low when submitted to abiotic stress conditions (heat and drought; Cao et al., 2014).

Two subtilases showing sequence homology with tomato P69C (XM_002275345.2 and XM_002275374.2) were up-regulated at 12 hpi and down-regulated at the other time-points (6 and 24 hpi), in the resistant cultivar Regent, and not amplified in the susceptible cultivar Trincadeira. In *S. tuberosum* leaves, was observed the up-regulation of the expression of three genes, homologous to these two grapevine subtilase genes, after inoculation with *Phytophthora infestans* or elicitation with DL-β-aminobutiric acid (BABA), a resistance gene inductor. This may suggest that these two genes could be pathogen induced and associated to the defense responses of resistant genotypes (Norero et al., 2016).

The subtilase XM_010660203.1 was previously identified by Figueiredo and co-workers (Figueiredo et al., 2008, 2012; Monteiro et al., 2013) as being up-regulated in the same pathosystem. This subtilase exhibited an high increase of expression in the resistant cultivar Regent at 6 hpi, but at 12 and 24 hpi the expression decreased, although it remained up-regulated (6 hpi: 324.63 ± 87.11; 12 hpi: 2.26 ± 0.38; 24 hpi: 2.00 ± 0.34). In the susceptible cultivar, Trincadeira, the expression of this subtilase increased during inoculation time-course (6 hpi: 1.08 ± 0.20; 12 hpi: 2.56 ± 0.77; 24 hpi: 4.15 ± 2.08). This subtilase may be considered as a strong *P. viticola* resistance associated candidate.

When comparing both incompatible (Regent) and compatible (Trincadeira) interactions it is clear that the increase in subtilase expression in Trincadeira presents a delay when compared to the resistant cultivar in which several subtilases are highly expressed 6 hpi (Figure 4). An early increase of expression of some subtilases in the incompatible interaction may be related to the successful establishment of a defense strategy against the invading pathogen.

It has been previously shown that the subtilase XM_010660203.1, annotated as a cucumisin (*VvSBT*1 group) was constitutively more expressed in the resistant cultivar Regent when compared to the susceptible cultivar Trincadeira, both in field and greenhouse conditions (Figueiredo et al., 2008, 2012). To access if the subtilases that are up-regulated at 6 hpi are also constitutively expressed in resistant genotypes we have conducted a qPCR expression analysis of these 6 subtilases (XM_003634104.2, XM_003634105.2, XM_002273159.3, XM_010659200.1, XM_010660203, XM_010649370.1) in several resistant (*V. labrusca, V. rupestris, V. rotundifolia, V. riparia*, and *V. candicans*) and tolerant (*V. sylvestris, V. vinifera* cv Regent) *Vitis* genotypes comparing to the constitutive expression in *V. vinifera* cv. Trincadeira (susceptible).

**Figure 5:**
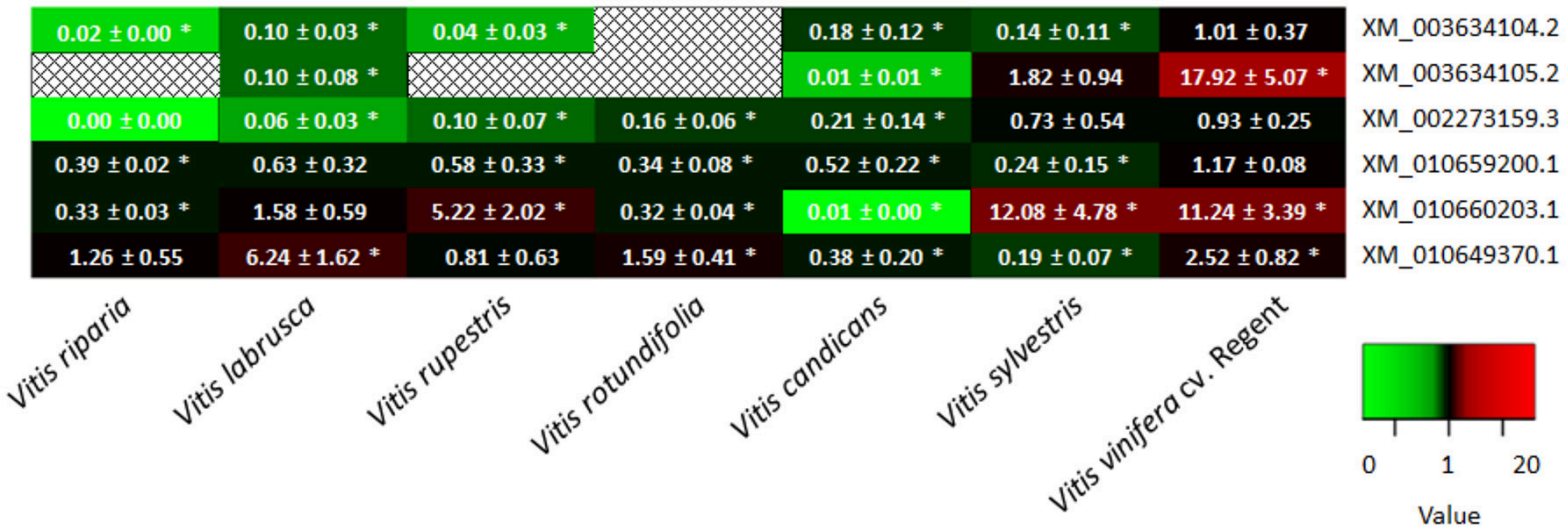
**Heatmap of the constitutive expression of 6 grapevine subtilases associated with ***P. viticola*** resistance (XM_003634104.2, XM_003634105.2, XM_002273159.3, XM_010659200.1, XM_010660203, XM_010649370.1)**. Each column indicates a *Vitis* specie and each row represents a subtilase gene. Green indicates lower expression, red indicates higher expression, black indicates no expression (see the color scale) and 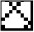 indicates no amplification. Asterisks (^*^) represent significant difference (*p* ≤ 0.05) between target and control samples (Mann–Whitney *U*-test; SPSS Inc., USA, V20).

None of the subtilases analyzed exhibited an up-regulated constitutive expression in comparison to Trincadeira (Figure 5). However, the subtilase XM_010660203.1, the cucumisin previously identified by our group, was more expressed in several resistant and tolerant species/cultivars (*V. labrusca*: 1.58 ± 0.59, *V. rupestris*: 5.22 ± 2.02, *V. sylvestris*: 12.08 ± 4.78, *V. vinifera* cv. Regent: 11.24 ± 3.39). Our results suggest that the expression of the majority of the grapevine subtilases analyzed may be induced by the pathogen, nonetheless the cucumisin XM_010660203.1 may be constitutively accumulated in some grapevine resistant genotypes, contributing to a rapid and strong increase of expression after pathogen inoculation.

## Conclusions

This work presents the identification and characterization of the grapevine subtilase family, comprising 82 genes (20 of each are intronless), after the grapevine genome reannotation (Vitulo et al., 2014). These genes present an uneven distribution along 15 of the 19 grapevine chromosomes and encode 97 putative *Vv*SBT proteins, due to alternative splicing. Phylogenetical analysis allowed the characterization of six groups (*Vv*SBT1–*Vv*SBT6) based on amino acid similarity.

Our results suggest that grapevine subtilases may exert several functions and that several grapevine subtilases may be potentially involved in pathogen defense, particularly to *P. viticola*. We have shown that several grapevine subtilases present sequence similarity with Arabidopsis SBT3.3 and tomato P69, some are located in the *P. viticola* resistance associated locis (*Rpv*) or are up-regulated during *P. viticola* infection. Also, the majority of subtilases are predicted to be located in the extracellular space which reinforces their putative role in the defense mechanisms against pathogens. XM_010660203.1, the cucumisin previously identified as possibly involved in the grapevine defense mechanisms (Figueiredo et al., 2008, 2012; Monteiro et al., 2013), presented the higher increase of expression after inoculation and it is constitutively expressed in several resistant grapevine genotypes, thus it may be considered a strong resistance-associated candidate. More studies must be conducted to define subtilase functions and their role on plant-pathogen interactions particularly in the grapevine resistance against *P. viticola*.

## Author contributions

AF conceived the study and planned the experiment. JF and MM performed the experiments; JF has done the bioinformatic analysis. GC and OP performed the phylogenetical analysis. AF, MS, RM, and JF performed data analysis. AF, JF, and MS wrote the manuscript. All authors have read and approved the manuscript.

### Conflict of interest statement

The authors declare that the research was conducted in the absence of any commercial or financial relationships that could be construed as a potential conflict of interest.

## Supplementary material

Supplementary Data 1 **Information about ***Vitis*** species and ***Vitis vinifera*** cultivars used in gene expression analysis of subtilase genes; R (resistant), T (tolerant), S (susceptible)**.

Supplementary Data 2 **Target and reference gene oligonucleotide sequences, amplicon length, amplification efficiency, annealing, and melting temperature are represented**.

Supplementary Data 3 **General features of grapevine subtilases**. Gene locus, nucleotide, and protein accessions, *Vv*SBT group, chromosomal location, number of intron and exons, protein predicted name, enzyme code, protein length, molecular weight, isoelectric point, domain, and Gene Ontology terms are represented.

Supplementary Data 4 **Targeting prediction of the 97 grapevine subtilases using either TargetP v1.1 or PredoTar v1.3**.

Supplementary Data 5 **Information about the top five proteins that interact with all of the 14 grapevine subtilases selected for expression studies**. STRING identifier, Uniprot ID, predicted identification and GO terms associated with the interactor protein are presented.
