# Supplementary material for: Revisiting Vitis vinifera Subtilase Gene Family: A Possible Role in Grapevine Resistance against Plasmopara viticola
Source: Front Plant Sci. 2016 Nov 25;7:1783. doi: 10.3389/fpls.2016.01783 (PMC5122586; doi:10.3389/fpls.2016.01783)
Supplement: Supplementary Data 1 — Information about Vitis species and Vitis vinifera cultivars used in gene expression analysis of subtilase genes; R (resistant), T (tolerant), S (susceptible). [file Table1.DOCX]

| Species | Type of Accession | Origin | Response to downy mildew |
| --- | --- | --- | --- |
| *V. labrusca* | Wild species | America | R |
| *V. rupestris* | Wild species | Southern and Western America | R |
| *V. rotundifolia* | Wild species | America | R |
| *V. riparia* | Wild species | North America | R |
| *V. sylvestris* | Wild species | America | T |
| *V. candicans* | Wild species | Southern America | R |
| *V. vinifera* cultivars | | | |
| Trincadeira | Cultivated grapevine | South Europe | S |
| Regent | Complex hybrid | Breeding | T |
