# Supplementary material for: Revisiting Vitis vinifera Subtilase Gene Family: A Possible Role in Grapevine Resistance against Plasmopara viticola
Source: Front Plant Sci. 2016 Nov 25;7:1783. doi: 10.3389/fpls.2016.01783 (PMC5122586; doi:10.3389/fpls.2016.01783)
Supplement: Supplementary Data 2 — Target and reference gene oligonucleotide sequences, amplicon length, amplification efficiency, annealing, and melting temperature are represented. [file Table2.DOCX]

| Adopted Identifier/ Accession Number | Primer sequence | Amplicon length (bp) | Amplification efficiency (E) | Ta (°C) | Tm (°C) |
| --- | --- | --- | --- | --- | --- |
| Reference genes (Monteiro *et al*. 2013) | | | | | |
| *EF1α*  (elongation factor 1-alpha)  *XM_002284888.2* | F: GAACTGGGTGCTTGATAGGC  R: ACCAAAATATCCGGAGTAAAAGA | 164 | 1.89 | 60 | 79.16 |
| *GAPDH*  (glyceraldehyde-3-phosphate dehydrogenase)  *XM_002263109.3* | F: TCAAGGTCAAGGACTCTAACACC  R: CCAACAACGAACATAGGAGCA | 226 | 1.99 | 60 | 80.95 |
| *UBQ*  (ubiquitin-conjugating enzyme)  *XM_002284161.3* | F: GAGGGTCGTCAGGATTTGGA  R: GCCCTGCACTTACCATCTTTAAG | 75 | 1.95 | 60 | 78.86 |
| *SAND*  (SAND family protein)  *XM_002285134.2* | F: CAACATCCTTTACCCATTGACAGA  R: GCATTTGATCCACTTGCAGATAAG | 76 | 1.93 | 60 | 79.16 |
| Target genes | | | | | |
| *XM_010663620.1* | F: CCATTATACACGACTCCCTT  R: TAACCGTCGCCACCAAACA | 88 | 1.96 | 58 | 77.48 |
| *XM_002273159.3* | F: CAAGCCCCATTAGCACAC  R: TTAGAATCAAGATCAAAGAAG | 87 | 1.92 | 56 | 72.95 |
| *XM_002284065.3* | F: CCAGTCCCTACAGTTTATC  R: ACACGCCGGAGTAGTTCTT | 120 | 1.96 | 58 | 83.45 |
| *XM_003634104.2* | F: GGCGTTCCCATTGCTTGAT  R: TTCCCTCGTCTTTGATTATTC | 111 | 1.96 | 58 | 76.59 |
| *XM_003634105.2* | F: CCTCCCAATGGAAAAATCTG  R: GGCTCATGCTATACAACAAG | 170 | 2.01 | 58 | 77.93 |
| *XM_002275345.2* | F: GCCGGAGGGTGGAGTTTTT  R: CATGCGTTCTTGCTGTTTTGA | 100 | 1.95 | 58 | 80.34 |
| *XM_002275374.2* | F: GGACGGCCTGCAACAACAA  R: ATGGCCCTCTTCATCAATAG | 86 | 1.90 | 58 | 79.28 |
| *XM_002275393.2* | F: TTGCATAAGGGGTCAGGGTT  R: CATTTCGCAGGTGGAGGTG | 134 | - | 60 | - |
| *XM_002275435.2* | F: TGACGGAGGAAGAAGTGAGA  R: GGGTGAATGCGTTGTTAGTA | 95 | 2.04 | 58 | 76.13 |
| *XM_010659200.1* | F: CAGCGAGTTTTAGTGATGAAG  R: GGGGTATGGAAGGAAGAGT | 172 | 1.96 | 58 | 79.58 |
| *XM_010649370.1* | F: GGGATATGGCCTGAGTCTGA  R: CAACGCGCACCGATTATTTT | 134 | 2.03 | 60 | 79.44 |
| *XM_002277863.3* | F: GTCCAACCTCACACTACC  R: GTTTTCCCATACCCTCGTC | 160 | - | 58 | - |
| *XM_002278414.3* | F: AAGGTGTACAAAGTGGCTAAA  R: CCTGGAAATGGAAAGATGTT | 102 | 1.89 | 58 | 75.39 |
| *XM_010660203.1* | F: AATCCTGGTGTTCTTGTGG  R: ATTAGGTAAAATGTTGTGCTTG | 73 | 2.05 | 58 | 72.11 |
