# Supplementary material for: Revisiting Vitis vinifera Subtilase Gene Family: A Possible Role in Grapevine Resistance against Plasmopara viticola
Source: Front Plant Sci. 2016 Nov 25;7:1783. doi: 10.3389/fpls.2016.01783 (PMC5122586; doi:10.3389/fpls.2016.01783)
Supplement: Supplementary Data 5 — Information about the top five proteins that interact with all of the 14 grapevine subtilases selected for expression studies. STRING identifier, Uniprot ID, predicted identification and GO terms associated with the interactor protein are presented. [file Table5.DOCX]

| Gene name  (gene identifier from STRING) | UniProt ID | Protein name | GO terms associated |
| --- | --- | --- | --- |
| VIT_10s0116g00330 | D7TR79 | Putative uncharacterized protein | **ER-associated ubiquitin-dependent protein catabolic process; positive regulation of RNA polymerase II transcriptional preinitiation complex assembly**; positive regulation of proteasomal protein catabolic process; nucleotide binding; ATP binding; TBP-class protein binding; proteasome-activating ATPase activity; proteasome regulatory particle, base subcomplex; nuclear proteasome complex; cytosolic proteasome complex |
| VIT_10s0116g00260 | D7TR76 | Putative uncharacterized protein | ER-associated ubiquitin-dependent protein catabolic process; **positive regulation of RNA polymerase II transcriptional preinitiation complex assembly;** positive regulation of proteasomal protein catabolic process; nucleotide binding; ATP binding; TBP-class protein binding; proteasome-activating ATPase activity; proteasome regulatory particle, base subcomplex; nuclear proteasome complex; cytosolic proteasome complex |
| VIT_00s0125g00170 | F6H2Q4 | Putative uncharacterized protein | protein targeting to peroxisome; fatty acid beta-oxidation; peroxisome organization; protein import into peroxisome matrix; nucleotide binding; ATP binding; ATPase activity, coupled; peroxisome; peroxisomal membrane |
| VIT_00s0540g00020 | F6HWU5 | Putative uncharacterized protein | carbohydrate metabolic process; chitin catabolic process; protein phosphorylation; **defence response**; hydrolase activity, hydrolyzing *O*-glycosyl compounds; **chitinase activity; protein kinase activity; protein serine/threonine kinase activity**; ATP binding; plasma membrane; plasmodesma; integral component of membrane |
| VIT_05s0102g00260 | F6HYR3 | Putative uncharacterized protein | **negative regulation of chromatin silencing; positive regulation of transcription from RNA polymerase II promoter**; nucleotide binding; chromatin binding; ATP binding; ATPase activity; histone binding; nucleus |
